# Yeast pheromone pathway modeling using Petri nets

**DOI:** 10.1186/1471-2105-15-S7-S13

**Published:** 2014-05-28

**Authors:** Abhishek Majumdar, Stephen D Scott, Jitender S Deogun, Steven Harris

**Affiliations:** 1 Department of Computer Science and Engineering, University of Nebraska, Lincoln, 256 Avery Hall, 68588-0115 Lincoln, USA; 2 Plant Pathology Departments, University of Nebraska, Lincoln, E126 BEAD, 68588-0660 Lincoln, USA

## Abstract

**Background:**

Our environment is composed of biological components of varying magnitude. The relationships between the different biological elements can be represented as a biological network. The process of mating in *S. cerevisiae *is initiated by secretion of pheromone by one of the cells. Our interest lies in one particular question: how does a cell dynamically adapt the pathway to continue mating under severe environmental changes or under mutation (which might result in the loss of functionality of some proteins known to participate in the pheromone pathway). Our work attempts to answer this question. To achieve this, we first propose a model to simulate the pheromone pathway using Petri nets. Petri nets are directed graphs that can be used for describing and modeling systems characterized as concurrent, asynchronous, distributed, parallel, non-deterministic, and/or stochastic. We then analyze our Petri net-based model of the pathway to investigate the following: 1) Given the model of the pheromone response pathway, under what conditions does the cell respond positively, i.e., mate? 2) What kinds of perturbations in the cell would result in changing a negative response to a positive one?

**Method:**

In our model, we classify proteins into two categories: core component proteins (set *ψ*) and additional proteins (set *λ*). We randomly generate our model's parameters in repeated simulations. To simulate the pathway, we carry out three different experiments. In the experiments, we simply change the concentration of the additional proteins (*λ*) available to the cell. The concentration of proteins in *ψ *is varied consistently from 300 to 400. In Experiment 1, the range of values for *λ *is set to be 100 to 150. In Experiment 2, it is set to be 151 to 200. In Experiment 3, the set *λ *is further split into *σ *and *ς*, with the idea that proteins in *σ *are more important than those in *ς*. The range of values for *σ *is set to be between 151 to 200 while that of *ς *is 100 to 150. Decision trees were derived from each of the first two experiments to allow us to more easily analyze the conditions under which the pheromone is expressed.

**Conclusion:**

The simulation results reveal that a cell can overcome the detrimental effects of the conditions by using more concentration of additional proteins in *λ*. The first two experiments provide evidence that employing more concentration of proteins might be one of the ways that the cell uses to adapt itself in inhibiting conditions to facilitate mating. The results of the third experiment reveal that in some case the protein set *σ *is sufficient in regulating the response of the cell. Results of Experiments 4 and 5 reveal that there are certain conditions (parameters) in the model that are more important in determining whether a cell will respond positively or not.

## Background

### Problem description

Yeasts are single celled microorganisms in the Fungi kingdom. *Saccharomyces cerevisiae *a particular species of yeast, has been widely studied in genetics and cell biology. *S. cerevisiae *has both asexual and sexual reproduction. Sexual reproduction takes place between two haploid cells of opposite types **a **and *α*. The process of mating is initiated by secretion of pheromone by one of the cells. Receptors on the opposite cell detect the presence of pheromone and initiates a series of protein-protein interactions within the cell that ultimately might facilitate mating. This series of protein-protein interactions in the cell is known as the yeast pheromone pathway. This pathway is well-studied. We have a working knowledge of how the pathway functions, the different proteins that take part in this pathway and their respective roles. However, several questions still remain unanswered. Our interest lies in one particular question: how does the cell dynamically adapt the pathway to continue mating under severe environmental changes or under mutation (which might result in the loss of functionality of some proteins known to participate in the pheromone pathway).

Our work attempts to answer this question. We first propose a model to simulate the pheromone pathway using Petri nets. We then analyze our Petri net-based model of the pathway to explore the following:

1 Given the model of the pheromone response pathway, under what conditions does the cell respond positively, i.e., mate?

2 What kinds of perturbations in the cell would result in changing a negative response to a positive one?

In our model, the "conditions" mentioned in Question 1 typically refer to the different edge weights between the different components of the Petri net-based pathway model. Different combinations of the values of the edge weights represent different environmental conditions faced by the cell. "Perturbations" mentioned in Question 2 refer to possible methods employed by the cell so that it can mate. We conjecture that one method might be the use of accessory proteins who otherwise are not so prominent in the pheormone pathway. Using appropriate amounts of proteins other than the core pathway component proteins can be a possible compensation method used by the cell to facilitate mating.

We generate a large number of networks and run experiments to identify "conditions" for a positive response. We employ decision trees [1] to analyse the effect of conditions on the pathway. The Petri net-based model gives us a set of conditions that allow us to predict whether the pathway responds positively. It also supports our conjecture about the possible use of other proteins as a compensation process to allow mating by giving positive instances of pheromone response for the networks that simulated the mentioned idea. Finally, we come across several rules or conditions that are highly consistent across all the simulated networks indicating their importance in determining the outcome of the networks.

### Petri nets

Petri nets were first proposed by Carl Adam Petri in 1962. Petri nets can be used for describing and modeling dynamic systems that can be characterized as concurrent, asynchronous, distributed, parallel, non-deterministic, and/or stochastic systems. The following is based on the discussion in [2,3].

A Petri net is a directed weighted bipartite graph with an initial state *M*0. The two types of nodes of the bipartite graph are called *places *and *transitions*, represented by circles and boxes respectively. There can be arcs from places to transitions as well as from transition to places. The arc weights are positive integers and absence of a weight implies unit weight. A marking is a vector that represents an assignment of a non-negative number of tokens (denoted by dots) in all places in a given Petri net. In a Petri net model of a dynamic system, conditions are represented by places and events by transitions.

#### Definitions

A Petri net is defined as a 5-tuple *π *= (*P, T, E, W, M*_0_), where *P *= {*p*_1_, *p*_2_, *.*., *p_m_*} denotes a set of places, *T *= {*t*_1_, *t*_2_, *.*., *t_n_*} represents a set of transitions, *E *⊆ (*P *× *T*) ∪ (*T *× *P*) defines flow relation in terms of arcs, *W *: *E → *{1, 2, 3, *..*.} is an arc weight function and *M*0: *P → *{0, 1, 2, *..*.} is the initial marking. It may be noted that the set of places *P *and the set of transitions *T *are totally disjoint sets.

Below we define some terminologies related to Petri nets. As stated earlier, a Petri net is a directed graph. A *preplace *of a transition *t*, is a place that is adjacent to *t*. The set of preplaces of *t *is denoted by **pre(*t*)**. Mathematically,

pre(t)={p|(p,t)∈E}.

Similarly, a *postplace *of a transition *t*, is a place adjacent from *t *and the set of postplaces of *t *is denoted by **post(*t*)**. Mathematically,

post(t)={p|(t,p)∈E}.

The *pre-transition *and *post-transition *concepts are defined similarly.

pre(p)={t|(t,p)∈E}

and

post(p)={t|(p,t)∈E}.

A set of rules defined below control the behavior of a Petri net model for simulating a dynamic system.

1 Let *w(p,t) *define the weight of an arc between *p *and *t*. We say that a transition *t *is *enabled *if each *p *∈ **pre(*t *) **has at least *w(p,t) *tokens.

2 If an event takes place, the corresponding enabled transition will fire otherwise not.

3 Let *| p | *denote the number of tokens in place *p*. Let *w(t,p) *define the weight of an arc between *t *and *p*. After a transition *t *has been fired the tokens will be updated as follows:

∀*p *∈ **pre**(t), |p| = |p| − w(p, t)

∀*ṕ *∈ **post**(*t*), *|ṕ| *= *|ṕ| *+ *w*(*t, p*)

**Figure 1:**
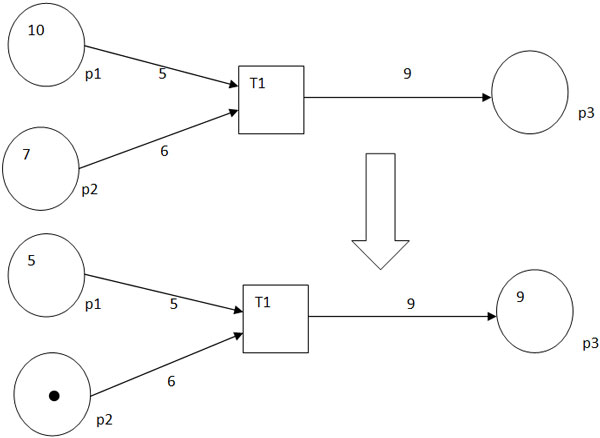
**A simple Petri net**. The top figure shows an example of a Petri net with 3 places *p1, p2 *and *p3 *and a transition *T1. p1 *has 10 tokens and *p2 *has 7 tokens, *w(p1,T1) *= 5, *w(p2,T2) *= 6 and *w(T1,p3) *= 9. When *T1 *fires, the Petri net will change as shown in the bottom figure. After *T1 *has fired, *p1 *has 5 tokens, *p2 *has 1 token and *p3 *has 9 tokens.

Figure 1 illustrates the workings of a Petri net.

### Related work

In this section we survey some of the papers in which a Petri net approach has been used to model biological networks.

Sackmann et al. [4] provide a systemic modeling method of signal transduction pathways in terms of Petri net components. The authors present a process of representing the following three different cases of a signal transduction model.

**Case 1**: A substance *A *does not lose its activity by interacting with a second substance *B*.

**Case 2**: A substance *C *triggers several reactions that are independent of each other.

**Case 3**: A substance changes state from being phosphorylated to being unphos-phorylated and vice versa.

Case 1 indicates phosphorylation reactions between different proteins in a network. Case 2 describes participation of a protein in multiple independent reactions. Both cases are implemented by using read arcs (bidirectional edges between places and transitions) in their Petri net representations. Case 3 indicates the different states of a protein, which is implemented in form of a sub-network. Having described these, the authors propose the following simple steps for representing a signal pathway. First, translate the biological components into logical strucures like conjunction, disjunction, exclusive disjunction and implication. Second, translate the logical structures in corresponding Petri net forms. Finally, assimilate the Petri net components to form a whole network. Our work uses the modeling approach used by this paper [4] and forms the basic structure of our model on the model provided in this paper [4].

Chaouiya [5] provides an overview of the different types of Petri net models available and their uses in modeling different types of biological networks. These include **Coloured Petri Net **(CPN), **Stochastic Petri Net **(SPN), **Hybrid Petri Nets **(HPNs) and **Hybrid Function Petri Nets **(HFPNs). Hardy and Robillard [6] also discuss the different types of Petri nets extensions used for analysis, modeling and simulation of molecular biology networks. They identify two categories of goals of Petri net biological modeling: **qualitative **and **quantitative **analysis. Qualitative analysis is the analysis of the different biological properties while quantitative analysis is the simulation of system dynamics. For quantitative analysis, a Petri net representation with sufficient modeling power should be chosen. For quantitative analysis of a biological system, kinetic parameters like reaction rates and stoichiometric quantities of reactants are necessary. Since no such data are available, we use the basic Petri net structure for our quantitative analysis. In the future, pending availability of data, we plan to upgrade our model to a HFPN or something similar. Monica et al. [7] demonstrate a generalized approach towards modeling and analysis of biological pathways using Petri nets.

### Yeast pheromone pathway

In this section, we describe the process of pheromone binding to its receptor on the cell surface and the subsequent effects of that phenomenon on the cell functionality. The summary description below is based on the description from [8,9]. The yeast mating process is initiated when a yeast cell detects the presence of pheromone secreted by a cell of the opposite sex. There are two cell types in yeast, called **a **and *α *that are analogous to egg and sperm cells of animals. The **a **and *α *cells can mate to produce an **a**/*α *cell. The cell **a**/*α *in turn undergoes meiosis to produce the haploid gametes (child cells) **a **and *α *cells. The pheromones produced respectively by **a **and *α *cells are *a-factor *and *α-factor*. An **a **cell contains the *α-factor *receptor Ste2 whereas an *α *cell contains the *a-factor *receptor Ste3. So **a **cells can mate with *α *cells only and vice-versa.

When either Ste2 and Ste3 binds with pheromone, its ability to bind with intracellular G protein complex is compromised. The G protein comprises three subunits known as Gpa1, Ste4 and Ste18. These subunits are commonly referred to as G*_α_*, G*_β_*, and G*_γ_*, respectively. The subunits G*_β _*and G*_γ _*units form a complex G*_βγ_*. If G*_α _*is bound to GDP then G*_βγ _*is bound to G*_α_*. When a pheromone binds to the receptor (Ste2 or Ste3), the receptor interacts with G*_α_*, causing it to replace its GDP with GTP. G*_α _*without its GDP cannot keep the G*_βγ _*complex bound to itself. As a result, the G*_βγ _*complex is liberated and goes on to interact with other proteins. Gradually, hydrolyzation of GTP bound to G*_α _*takes place. G*_α _*then binds back and inhibits the G*_βγ _*complex in absence of pheromone.

The liberated G*_βγ _*complex, activates four protein kinases linked in form of a cascade. Protein Ste5 acts as a scaffold to hold the three other proteins Ste11, Ste7 and Fus3 in place. These three proteins activate each other in series by phosphorylation. So an activated Ste11 phosphorylates Ste7 which becomes active and in turn phosphorylates Fus3. The activated Fus3 then enters the nucleus. The Ste11 at the top of the kinase is activated by a protein Ste20. The protein Ste20 itself becomes activated when it is in the plasma membrane where it is phosphorylated by Cdc42 which is a membrane associated monomeric GTPase.

Activated Fus3 plays an important role in both cell cyle arresting as well as the transcription of genes. Activated Fus3 phosphorylates protein Far1 which blocks the cell cycle in G1 phase, to prepare for mating. Fus3 in the nucleus activates the transciption factor Ste12. Normally, Ste12 is inhibited by proteins Dig1 and Dig2, when pheromone signal is not present. Due to pheromone signalling, activated Fus3 phosphorylates proteins Dig1 and Dig2 which in turn release Ste12. The Ste12 is then free to bind and promote the transcription of **a**-specific genes (***a-sgs***) and *α*-specific genes (*α-sgs*).

The process of growing projection called a schmoo between cells, is an important feature of mating. The cell surface which faces the highest concentration of pheromone contains the most activated receptors. So the concentration of activated G*_βγ _*is highest here. The G*_βγ _*complex engages proteins for the formation of the shmoo. Far1 engages the proteins Cdc42, Cdc24 and Bem1, to promote schmoo after binding to G*_βγ _*complex. Cdc24 activates Cdc42, which together with Bem1 recruit proteins to promote cell membrane growth such as Bni1 and others. A mating process can succeed or fail. However yeast cells have a mechanism to re-enter the cell cycle using negative feedback loops.

## Method

### Model

**Figure 2:**
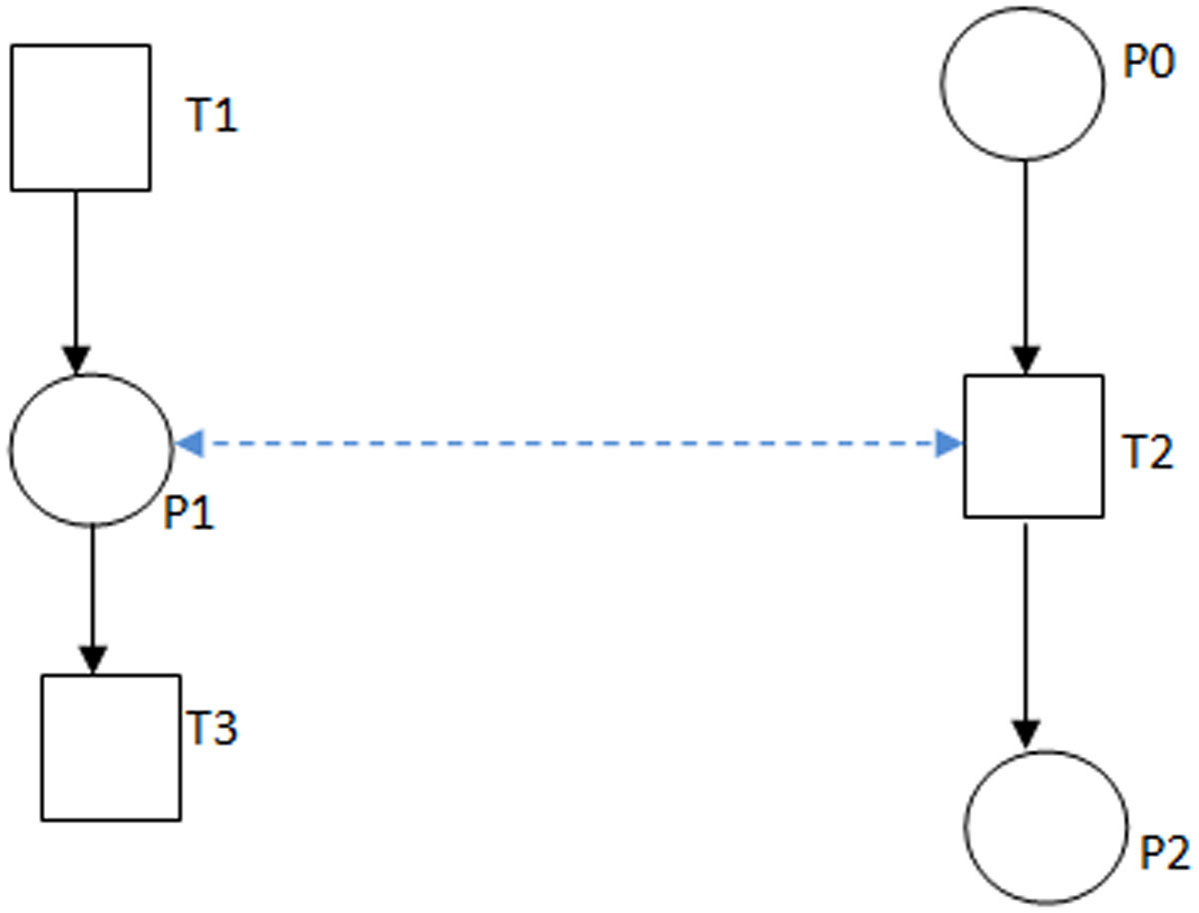
**The whole pheromone network**. This figure illustrates the full structure of our representation of the pheromone pathway.

We use Petri nets to model the pheromone response pathway. We represent each protein as a place in the Petri net and each interaction as a transition. Using this representation, the full pathway can be obtained by combining these individual reaction representations. Such a model is already available in the paper by Sackmann et al. [4] We base our model on this avaiable network structure [4] and make several changes to suit our approach. Motivating the first change, we know that the reaction between two or more proteins takes place if the strength of their interaction (kd value) exceeds a certain threshold. A traditional Petri net does not allow one to implement this concept. In our approach we transform the preplaces of all transitions to a single place (colored red in our structure Figure 2), which has inputs from different reactant places. We add a dummy transition to each reactant place. Only for transitions with Ste-type proteins as pre-places are left unchanged. The benefit of having a single pre-place to a transition that originally required several pre-places is that it emphasizes the notion of weighted cumulative concentration of the reactants.

In our second change to Sackmann et al.'s model we add more proteins that are known to interact with various component proteins of the pheromone pathway. We obtain these additional proteins from the yeast genome database [10]. The steps followed are described below. First, for each of the 20 protein components in the core pathway, namely Ste5, Ste11, Ste7, Ste20, Ste50, Fus3, Dig1, Dig2, Ste12, Sst2, Far1, Cdc24, Cdc42, Bem1, Ste2, Ste3, Ste4, Ste18, GPA1 and Tec1, we list all proteins that are known to interact with them physically. From this list we select only those proteins that are known to participate in the pheromone pathway reactions. Table 1[4] gives a list of all the protein components and their symbols used in our model. Table 2[4] gives a list of all the transitions, their symbols and biological reactions that they represent. Table 3 lists the 37 new proteins, which we have added to the pathway.

**Table 1:** Places of the model [4]

Symbol	Place Name	Biological species
p1	alpha-factor	pheromone released by an MAT*α *cell in the surroundings
p2	Ste2_receptor	mating pheromone receptor of the modelled MATa cell
p3	receptor_factor_complex	complex consisting of the *α *factor and the Ste2 receptor
p4	receptor_complex	the above named complex is activated by a conformation change
p5	trimer_bound_to_receptor	heterotrimeric G protein, which is coupled to the Ste2 receptor
p6	G_alpha_GTP	dissociated G*α *subunit (exchange of GDP to GTP in this monomer)
p7	G_beta_gamma_dimer	G-protein G*_βγ _*subunits in a dimer form
p8	Cdc24	Cdc24, i.e., guanine nucleotide exchange factor of Cdc42
p9	Cdc42(at pm)	Cdc42 located at the plasma membrane
p10	Ste20	protein kinase Ste20
p11	Ste5(scaffold)	Ste5, acting as a scaffold protein
p12	Ste5/Ste11	protein complex consisting of ste7 and Fus3
p13	Fus3	MAP kinase Fus3
p14	Ste7/Fus3	protein complex consisting of Ste7 and Fus3
p15	MAPK_complex	MAPK complex consisting of Ste5,Ste11,Ste7 and Fus3
p16	Ste20_at_pm	Ste20 located at the plasma membrane, i.e., near the MAPK complex
p17	complex2	as complex1, but Ste11 is activated additionally
p18	complex3	as complex2, but Ste7 is activated additionally
p19	complex4	as complex3, but Fus3 is activated additionally
p20	Fus3PP	dissociated Fus3 in the activated form
p21	complex_without_Fus3	as complex4, but without Fus3
p22	repr_complex	complex containing Ste12 repressed by Fus3 or Kss1 and Dig1/Dig2
p23	Dig1/Dig2	Ste12 inhibitors, i.e., cofactors for the repression
P24	free_Ste12	Ste12 released out of the repression complex
p25	Ste12	activated transcription factor Ste12
p26	Msg5	phosphatase Msg5 being able to deactivate Fus3 or Kss1
p27	Fus3_dephos	deactivated Fus3
p28	other_genes	pheromone regulated genes encoding mating related cell responses
p29	Bar1_in_nucleus	synthesised protease Bar1 located in the nucleus
p30	Bar1	Bar1 secreted in the cell environment
p31	inactive_Far1	synthesised Far1 located in the nucleus in an inactive form
p32	Far1	Far1 activated by phosphorylation
p33	Far1_in_cytosol	active Far1 located in the cytosol
p34	Sst2_in_nucleus	synthesised Sst2 located in the nucleus in an inactive form
p35	phos_Sst2	Sst2 activated by phosphorylation
p36	Sst2	active Sst2 located in the cytosol
p37	inactive_component	complex labelled for degradation by phosphorylation
p38	phos_Kss1	MAP kinase Kss1 activated by phosphorylation
p39	unphos_Kss1	inactive Kss1
p40	Akr1	protein Akr1 located at plasma membrane
p41	Yck1/Yck2_at_pm	kinases Yck1/Yck2 being able to label the Ste2 for degradation
p42	inactive_receptor	receptor labelled for ubiquitination and endocytosis
p43	Ste11	protein kinase Ste11
p44	Ste50	protein kinase Ste50
p45	Bem1	protein Bem1
p46	Ste7	protein kinase Ste7

**Table 2:** Transitions of the model [4]

Symbol	Transition Name	Biological Event
t1	MATalpha_cell(surroundings)	MAT*α *cell secretes its mating pheromone
t2	binding_factor_to_receptor	*α*-factor binds to the Ste2 receptor
t3	receptor_synthesis	synthesis of the cell surface Sst2
t4	receptor_conformation_change	conformation change of the receptor
t5	division(in_alpha_subunit:GDP)*→*GTP	dissociation of the G*α *subunit of the G-protein
t6	hydrolysis_GTP*→*GDP	hydrolysis reassociates G*α *with G*βγ*
t7	interact_through_Far1	G*βγ *interacts Far1 transmitted with Cdc24
t8	Cdc42:GDP*→*GTP	Cdc24 activates Cdc42
t9	active_Cdc42_constitutive_at_pm	constitute active Cdc42 attending the processes
t10	Ste20_input	source of Ste20
t11	Ste20_activated	Cdc42 at plasma membrane and Bem1 activates Ste20
t12	Ste5_input	source of Ste5
t13	Ste5_binds_Ste11	Ste5 binds Ste11
t14	Fus3_synth	synthesis of kinase Fus3
t15	Fus3_binds_Ste7	Ste7 binds Fus3
t16	complex-formation	Ste5/Ste11 binds Ste7/Fus3
t17	Ste20_phos_Ste11	phosphorylation of Ste11 by Ste20
t18	Ste11_phos_Ste7	phosphorylation of Ste7 by Ste11
t19	Ste7_phos_Fus3	phosphorylation of Fus3 by Ste7
t20	Fus3PP-release	release of activation Fus3 out of the MAPK complex
t21	binding_free_Fus3	remaining MAPK complex binds Fus3
t22	Ste12_inhibit_phos	phosphorylation of Ste12 inhibitors Dig1/Dig2 by Fus3PP
t23	Ste12-release	release of Ste12 out of the repression complex
t24	Ste12_phos	phosphorylation of Ste12 by Fus3PP
t25	transcr_activation	transcription activation of pheromone regulated genes
t26	Fus3PP_dephos	dephosphorylation of Fus3PP by Msg5
t27	repression_through_Fus3	Ste12 repression through inactive Fus3 and Dig1/Dig2
t28	cell_fusion	processes leading to the fusion of the two haploid cells
t29	transport_out_of_cell	Bar1 transport into the cell environment
t30	factor_destruction	Bar1 transmitted destruction of the *α*-factor
t31	Far1_phos	phosphorylation of Far1 by Fus3PP
t32	cell_cycle_arrest_in_G1	Far1 caused arrest in the cell cycle phase G1
t33	transport_out_of_cell	Bar1 transport into the cell environment
t34	Sst2_phos	phosphorylation of Far1 by Fus3PP
t35	transport_out_of_nucleus	Sst2 transport out of the nucleus
t36	accelerated_hydr_GTP*→*GDP	accelerated hydrolysis reassociates the G-protein
t37	Ste11_neg_phos	Fus3PP labels the MAPK complex at Ste11 for degradation
t38	degradation	degradation of the MAPK complex
t39	Ste7_neg_phos	Fus3PP labels the MAPK complex at Ste7 for degradation
t40	Ste7_phos_Kss1	phosphorylation of Kss1 by Ste7
t41	accelerated-dephos-Kss1	deactivation of phosphorylation Kss1 by Fus3PP
t42	Kss1_dephos	dephosphorylation of phosphorylated Kss1 by Msg5
t43	repression_through_Kss1	Ste12 repression through inactive Kss1 by Msg5
t44	tech_input	techinal:the repressed Ste12 complex assumed to be present
t45	Akr_synthesis	synthesis of Akr1
t46	Akr1_binds_Yck1/Yck2	Akr1 binds Yck1/Yck2
t47	receptor_phos	labelling of Ste2 for degradation
t48	ubiquit_endocytosis	ubiquitination and endocytosis of the receptor

**Table 3:** Additional interacting proteins *λ*

Symbol	Protein Name	Neighboring Components
a1	CBK1	STE5,STE20,STE50
a2	PTC1	STE5,STE20
a3	CLA4	STE11,CDC24,CDC42,BEM1
a4	DSE1	STE11,STE4
a5	HOG1	STE11,STE7,STE50
a6	PBS2	STE11,BEM1
a7	SHO1	STE11,STE20,STE50,SST2,CDC24
a8	SPA2	STE11,STE7
a9	SPH1	STE11,STE7
a10	RGA2	STE20,CDC24,CDC42,BEM1
a11	CLN2	STE20,DIG1,DIG2,FAR1
a12	ENT2	CDC24,STE20
a13	EXO84	STE20,BEM1
a14	BOI1	STE20,FUS3,DIG1,DIG2,CDC24,CDC42,BEM1
a15	CDC28	STE20,FAR1,BEM1
a16	GIC1	STE50,CDC42
a17	GIC2	STE50,CDC24,CDC42
a18	BN1	FUS3,CDC42
a19	MPT5	FUS3,SST2
a20	KDX1	TEC1,DIG1,DIG2,STE12
a21	KSS1	STE5,STE11,STE7
a22	WHI3	TEC1,SST2,STE2
a23	BZZ1	DIG1,DIG2
a24	HMLALPHA1	DIG1,DIG2,STE12
a25	HYM1	DIG1,DIG2
a26	YCK2	DIG2,STE3
a27	RSR1	CDC42,BEM1,CDC24
a28	SEC15	CDC24,BEM1
a29	EXO70	CDC42,BEM1
a30	SEC3	CDC42,BEM1
a31	RHO1	BEM1,STE4
a32	SEC6	BEM1,STE2
a33	AKR1	BEM1,STE2
a34	DIB1	STE7,DIG1
a35	YHR131C	STE20,FUS3
a36	BDF2	STE20,FUS3
a37	SAS10	STE20,FUS3
a38	RBS1	DIG1,DIG2
a39	YJR003C	DIG1,DIG2
a40	AXL2	CDC24,CDC42,BEM1
a41	BEM4	STE20,CDC24,CDC42

**Figure 3:**
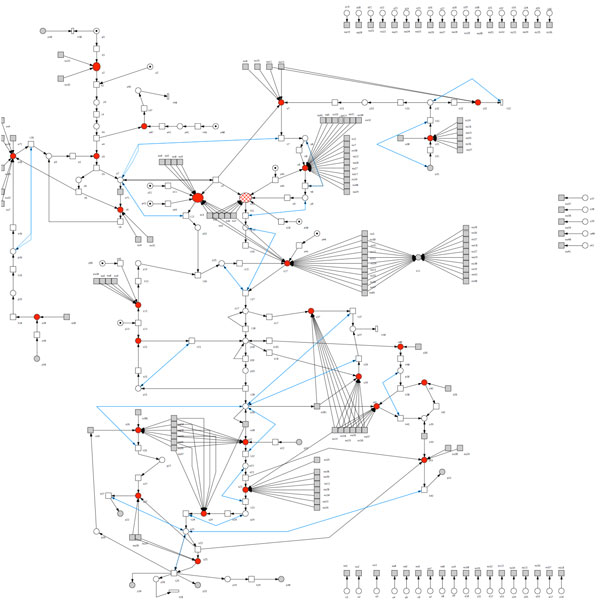
**Example of regulatory edge**. In Figure 3, reaction *T*1 produces compound *P*1, which participates in reaction *T*2. Protein *P*0 participates in reaction *T*2 which in turn produces *P*2. In the figure the bidirectional edge (blue edge) between *P*1 and *T*2 is a *regulatory edge *that makes sure that *T*2 will not fire until *P*1 is produced by *T*1 irrespective of the amount of *P*0 present.

We take these 37 additional proteins and add them to our network structure in the following manner. For each protein *i *which has *j *as a neighboring protein, we make *i *participate in all the reactions in which *j *is a reactant. In terms of our model, *i *becomes a preplace to all the post-transitions of *j*. After adding the additional proteins we add *regulatory edges *(dashed blue line) in Figure 3 in the network to control the order in which transitions may fire in the network. We define *regulatory edges *as bidirectional egdes of weight one between a place and a transition which makes sure that the transition cannot fire until that place has at least one token. Bidirectionality ensures that the token content of the place is not affected by the firing of the transition. We illustrate this with the help of Figure 3. The full pathway representation is shown in Figure 2.

### Experimental setup

We have developed a Java program that generates instances of the model described in the previous section. Due to the absence of real world data about the kd values for the different reactions in the pathway, we generate all the edge weights in our model randomly. The range of values for the edge weights used in our experiments is between 1 and 100 (extremities included). The places representing the components *α*-factor, Ste2-receptor, Ste20, Ste5, Fus3, Akr1, Ste11, Ste7, Ste50 and Bem1 were provided with initial concentration values. Let *ψ *represent the set of these 10 core component proteins. All places representing the additional components were also provided with initial concentration values. Let *λ *represent the set of all 41 additional protein components in our model. For a given value of concentration of all the proteins in sets *ψ *and *λ*, the network is simulated. It is checked whether the transition producing Ste12 has fired or not. If yes, then the pathway has responded successfully and the resultant concentration values of the different proteins are recorded.

### Experiments

We use the ANDL (Abstract Net Definition Language) [11] description of a Petri net (obtained from Snoopy [12]) to generate random networks for the model. We randomly generate the kd values for the different reactions in the pathway. To simulate the pathway, we carry out three different experiments. For the yeast pheromone pathway, apart from the structure of the pathway, exact kd values for each reaction are not known. From the literature, it can be seen that some experiments do provide possible kd values for some reactions. However, such values cannot be used in a generic way because they are specific to particular experiments. We assume that the value of kd for each reaction lies in the set {1, 2, . . . , 100} [13]. In absence of real life data, we generate the kd value for each reaction randomly from the set {1, 2, . . . , 100}, i.e., we assign weights to the different edges in the network structure randomly from {1, 2, . . . , 100}. The values allowed for each edge are discrete as Petri nets do not allow interchange of fractional tokens. For each experiment, the values of concentration allowed for the proteins in set *ψ *is from {300, 301, . . . , 400} (since Petri nets only allow integer number of tokens to be exchanged). The set of values for proteins in set *λ *vary in each experiment. Also, in the simulation, values of all elements in each set *ψ *or *λ *change together. That is, when one protein in set *ψ *has a concentration value of 300 (say), all the other proteins in *ψ *are also given the same value. The same is done for *λ*. In the rest of the paper when we say "value for *ψ*" we mean the value of the initial concentration of the proteins in *ψ*; similarly, "value for *λ*" means the value of the initial concentration of the proteins in *λ*. In a biological context, when we are simulating a network with its randomly generatd edge weights, the edge weights represent different conditions the cell is subjected to while it tries to respond to the pheromone.

1 **Experiment 1**: The range of values of initial concentration for the proteins in *λ *is set to be between 100 and 150. We generate 14443 networks and check for the response of the pathway in each of them. The networks generated represent a good sampling but not all possible scenarios. The objective of Experiment 1 is to identify conditions (i.e., different edge weights) under which the cell responds positively to the pheromone pathway.

2 **Experiment 2**: We take the 14443 networks generated in Experiment 1, and isolate the networks based on their responses. The ones which gave a negative response are put in set *neg*, while the ones with a positive response are put in set *pos*. We again run the simulation on each of the networks in *neg *but now we let the values of concentration of the proteins in *λ *to be from {151, 152, . . . , 200}. The objective of Experiment 2 is to test if the cell can overcome the conditions which made it respond negatively in Experiment 1, by using more concentration of proteins in the set *λ*.

3 **Experiment 3**: We partition the set *λ *into sets *σ *and *ς *such that *λ *= *ς *∪ *σ *and *σ∩ς *= *∅*. The proteins CBK1, PTC1, DSE1, SPA2, SPH1, MPT5, KDX1, HYM1, DIB1, YHR131c, BDF2, SAS10, RBS1 and YJR003c from *λ *are placed in *σ*. The rest are placed in *ς*. We propose that the proteins in *σ *contribute more to the pheromone pathway than the ones in *ς *and hence consider them to be more significant in their role in the pathway [13]. To simulate this, we let the values for the concentration of those proteins to be from {151, 152, . . . , 200}. For the proteins in *ς*, the range is set to be {100, 101, . . . , 150}. For all networks in set *pos *from Experiment 2, we run the simulation and look for positive responses.

## Results and discussion

1 **Result of experiment 1**: From the 14443 generated networks, 14187 networks gave a negative response. That is, for all 5151 combinations of values of initial concentrations of the proteins in *ψ *and *λ*, in each of the above mentioned 14187 networks, the transition which results in the production of protein Ste12 did not fire. The remaining 256 networks gave a positive response. The output of networks giving a positive response are of two types.

(a) A network starts giving a positive response when the value for *ψ *is ≥ some value *x *∈ {300, 301, . . . , 400} and the value for *λ *≥ 100. For instance, if a network starts giving a positive response when the value for *ψ *is 374 and the value for *λ *is 100, it means that, for this particular network with its set of edge weights (henceforth called a *configuration *of the network), as soon the as value for *ψ *exceeds 374, it will give a positive response irrespective of the concentrations of the proteins in *λ*.

(b) A network starts giving a positive response when the value for *ψ *is ≥ some *x *∈ {300, 301, . . . , 400} and the value for *λ *exceeds some value *y *∈ {101, . . . , 150}. For instance, if a network starts giving a positive response when the value for *ψ *is 374 and the value for *λ *is 105, that means, for this particular network with its corresponding configuration to respond positively, it is not sufficient that the values for just *ψ *become 374. The value for *λ *also needs to exceed value 105.

2 **Result of experiment 2**: Out of the 14187 networks, 13779 networks still gave a negative response. The remaining 408 networks responded positively. That is, out of these 408 networks, each one started giving positive responses when the value for *ψ *is ≥ some value *x *∈ {300, 301, . . . , 400} and the value for *λ *exceeds some value *y *∈ {151, . . . , 200}. That is, by increasing the initial concentration level of the proteins in *λ*, these networks changed their response from negative in Experiment 1 to positive in this experiment. So this means for these 408 networks, the additional proteins in *λ *play a significant role in deciding how the network responds to the pathway. Changing a prior negative response to a positive one indicates that these proteins might potentially be able to compensate for the lack of some of the core protein components in the pathway if present in sufficient amount.

3 **Result of experiment 3**: Based on the output of each network, the networks can be classified into three categories.

(a) The class CS (Class Same) represents those networks that gave positive responses in both Experiments 2 and 3 using the same combination of values for its proteins. That is, if a network gave a positive response in Experiment 2 with values *x *as the value for *ψ *and *y *as the value for *λ*, it gives a positive response in Experiment 3 as well with the same combination of values; *x *as the value for *ψ *and *y *as the value for *σ*. For instance, if a network in CS gave a positive response in Experiment 2 when the value for *ψ *exceeded 374 and the value for *λ *exceeded 105, it gives a positive response in Experiment 3 when the value for *ψ *exceeded 374 and the value for *σ *exceeded 105. Out of the 408 networks (from *pos*) used for this experiment 67 of them were placed in class *CS *because of their output.

(b) The class CD (Class Different) represents those networks which gave positive responses in both Experiments 2 and 3 but using the different combination of values for its proteins. For instance, if in Experiment 2, the network had initial concentration values *x *for the proteins in *ψ *and *y *for those in *λ*, in Experiment 3 it has *x *as initial concentration value for proteins in *ψ *and *z *for those in *σ *where *y *≠ *z*. Such a network is placed in class *CD*. Out of the 408 networks, 60 of them were placed in class *CD*.

(c) The class CN (Class Negative) represents those networks that gave positive responses in Experiment 2 but now give negative responses in Experiment 3. 281 networks from set *pos *gave negative response and were placed in class *CN*.

### Interpretation of results

1 Experiment 1: Networks that give a positive response when the value for *ψ *is ≥ some value *x ∈ *{300, 301, . . . , 400} and the value for *λ *≥ 100 indicate that for these networks with their corresponding set of edge weights, the additional proteins in *λ *play no significant role in controlling their responses. The response is based solely on the initial concentration of the core component proteins in *ψ*. Networks that start giving a positive response when the value for *ψ *is ≥ some value *x *∈ {300, 301, . . . , 400} and the value for *λ *≥ some *y *where *y *∈ {101, . . . , 150} indicate that for these networks with their given configuration, depend on the additional proteins in *λ *for modulating their response to the pheromone pathway. That is, for these networks it is the additional proteins in *λ *which makes the response positive when the value for *ψ *is not sufficient. In a biological context, such networks show that under those conditions the yeast cell uses the proteins in *λ *to facilitate mating. Networks with negative responses indicate the conditions under which a cell will not mate for any combination of initial concentrations of its different proteins.

2 Experiment 2: The 408 networks that start responding positively indicate that the amount of concentration for proteins in *ψ *or *λ *allowed in Experiment 1 was not sufficient for them to give a positive response. So the cell compensated by using more amounts of those additional proteins in *λ *to facilitate mating. The increase of the range of allowable values for *λ *by us simulate the cell using more concentration of proteins than what it was using in Experiment 1. These networks support our hypothesis that the cell probably uses one or more additional proteins to respond favorably to the pheormone pathway when it is unable to produce a positive response using just the core component proteins.

3 Experiment 3: Networks in class *CS *tell us that for these networks with their corresponding configurations the set of proteins in *σ *play a more significant role in the pheromone pathway than the rest of the proteins in *ς*. This indicates that a particular network does not require higher concentrations of all the proteins in *λ *to change its response from negative to positive. The proteins in *σ *are alone capable of doing so. So these networks represent conditions under which the cell rely more on the proteins in *σ *than those in *ς *to facilitate a change in response from negative to positive.

### Analysis of experiments

#### Development of decision trees

In order to identify reasons that might determine whether a network responds positively or negatively, we use *decision trees *to identify important attributes in the network. Decision trees are learning methods which are used to classify instances based on their attribute values. Each internal node is a test of some attribute and the leaves represent different classes. The tree is supposed to reflect the conditions for positive response and to identify the attributes that influence this positive response. It also provides an easy way of visualizing the impact of the attributes. We quantify the importance of each attribute by their distance from the root. We use Weka 3.6 (Waikato Environment for Knowledge Analysis) [14] software for this purpose. We consider each edge in the network as its different attributes.

1 **Experiment 4**: We take the output of Experiment 1 and divide the output into two classes *P *and *N*. Networks that give postive responses are put in class *P *while the ones with negative response are put in class *N*. For each network, each of its edge weights is listed as an attribute for that network followed by its class *P *or *N*. From the results of Experiment 1, it is seen that the number of networks responding positively is very small compared to those responding negatively. For this reason we derive 3 different decision trees from 3 sets of data inputs D1, D2 and D3. D1 has equal numbers of positive and negative networks i.e. 256 postive networks and 256 negative networks. D2 has 256 positive networks and 750 negative networks. D3 has 256 positive networks and 1024 negative networks. All the negative networks are selected randomly out of the set of 14443 negative networks obtained from Experiment 1. Once the list is completed for all the datasets, it is given to the J48 decision tree program implemented by Weka 3.6 [14] as an input. A 10-fold cross validation [15] is carried out to get a better estimate of the performance of the decision tree for each data set. We compare the different nodes at each level of a decision tree across all the ten trees generated by Weka. This is done to look for attributes which get tested most often (in more than 5 out of 10 trees) at the same level and the corresponding values against which they are tested. We look at the first four levels starting from the root of each tree. We use three different datasets to ascertain the influence of increasing number of labelled negatives in the data on the accuracy and attribute selection of each tree.

2 **Experiment 5**: We take the output of Experiment 2 and divide the output into two classes *P *and *N *based on their response as mentioned in Experiment 4. We create a dataset by listing each edge weight of each network followed by their corresponding classes. Again, three datasets are created E1, E2 and E3. E1 has equal instances of positive and negative networks, i.e., 408 postive networks and 408 negative networks. E2 has 408 positive networks and 1000 negative networks. E3 has 408 positive networks and 2000 negative networks. All the negative networks are selected randomly out of the set of 13779 negative networks obtained from Experiment 2. Each dataset is fed to J48 in Weka and 10-fold cross validation is carried out. We compare the nodes at each level across all the 10 trees for the first 4 levels for look for common attributes that get tested often (in more than 5 out of 10 trees) at the same level across all trees.

3 **Experiment 6**: We divide the output of Experiment 3 in into 3 classes *CS, CD *and *CN*, based on their individual responses. These 3 classes are the same ones that we described in Experiment 3. Once all the networks have been classified, a data set describing the attribute and class of each network is created as mentioned above. The data set is fed to J48 and a 10-fold cross validation is carried out. We compare the nodes at each level across all the 10 trees for the first 4 levels for look for common attributes that get tested often (in more than 5 out of 10 trees) at the same level across all trees.

#### Interpretation of trees

Tables 4 and 5 give the classification results of the decision trees developed in Experiment 4 and Experiment 5, respectively. In both experiments, as the number of negative networks increases in a dataset, the classification accuracy of predicting a negative response also increases, which is expected to happen. Tables 6 and 7 list the most commonly compared nodes across 10 decision trees for Experiments 4 and 5, respectively. They also indicate the corresponding values for each attribute, i.e., the weight of the corresponding edges in the model. In the tables the median values of the attributes from among all the trees have been listed. Level 1 is the root node of the tree and subsequent levels refer to nodes at lower levels. The impact of a node depends on its proximity to the root node. Thus in both tables the levels arranged in decreasing order of importance is *Level*1 *> Level*2 *> Level*3 *> Level*4. Table 8 indicates the biological meaning of these nodes in the pheromone pathway.

**Table 4:** Experiment 4 decision tree classification accuracy

Dataset	Positive network	Negative network
	**Accuracy(%)**	**Accuracy(%)**

D1	70.70	67.96
D2	49.21	84.40
D3	44.14	86.04

**Table 5:** Experiment 5 decision tree classification accuracy

Dataset	Positive network	Negative network
	**Accuracy(%)**	**Accuracy(%)**

E1	63.72	64.71
E2	47.79	79.10
E3	31.37	86.90

**Table 6:** Hierarchy of nodes in experiment 4

Dataset	Level 1	Level 2	Level 3	Level 4
D1	e4TOs5, 17	t2TOp3, 27	s2TOt2, 86	s7TOt7, 63.5
		s5TOt5, 26		ea17TOs11, 14
D2	t2TOp3, 43	e4TOs5, 41	t2TOp3, 25	ea12TOs8, 53
			t4TOp4, 13	p3TOt4, 14
				s2TOt2, 88
D3	t2TOp3, 40	e4TOs5, 21	t4TOp4, 13	p3TOt4, 25
				s2TOt2, 69

**Table 7:** Hierarchy of nodes in experiment 5

Dataset	Level 1	Level 2	Level 3	Level 4
E1	e4TOs5, 11	s5TOt5, 35	t4TOp4, 12	p3TOt4, 21
		t2TOp3, 42		s2TOt2, 93
E2	t2TOp3, 47	s5TOt5, 39	s2TOt2, 55	t4TOp4, 15
		e4TOs47, 57	p4TOe4, 60	p3TOt4, 26
			e4TOs5, 15	
			t4TOp4, 15	
E3	t2TOp3, 48	s5TOt5, 39	s2TOt2, 55	p3Tot4, 37
		e4TOs5, 14	t4TOp4, 15	e4TOs47, 44
				e4TOs5, 21
				t4TOp4, 26

**Table 8:** Impact nodes and their meanings

Node	Biological meaning
e4TOs5	Amount of receptor_complex contributing to the reaction: dissociation of the G*α *subunit of G-protein
s5TOt5	kd value required for reaction: dissociation of the G*α *subunit of G-protein
t4TOp4	Amount of receptor_factor_complex formed due to conformation change of receptors
p3TOt4	Minimum concentration of receptor-factor complex required for conformation change of the receptor
p4TOe4	Minimum concentration of receptor_complex required to participate in the pathway
t2TOp3	Amount of receptor-factor complex formed due to binding of *α *factor to receptor
s2TOt2	kd value required for reaction: *α*-factor binds to the Ste2 receptor
s7TOt7	kd value required for reaction: G*βγ *interacts Far1 transmitted with Cdc24
e4TOs47	Amount of receptor_complex contributing to the reaction: labelling of Ste2 for degradation
ea17TOs11	Amount of additional protein GIC2 contributing to the reaction involving activation of Ste20
ea12TOs8	Amount of additional protein ENT2 contributing to the reaction: activation of Cdc42 by Cdc24

## Conclusion

The simulation experiments reveal three kinds of results. From the results of Experiment 1 we learn about different conditions under which a cell will respond to a pheromone. There are some conditions under which a cell does not respond at all. However if a cell responds positively, there are two possible methods for its response: either the response is solely dependent on the initial concentrations of its core component proteins in *ψ *or the response is to some extent dependent on the concentration of the (additional) proteins in *λ *as well. In Experiment 2 we look for possible changes that a cell might adopt so that it can mate in conditions under which it responded negatively in Experiment 1. This is simulated by allowing the cell to utilize larger concentrations of proteins in *λ*. The results reveal that the cell can overcome the detrimental effects of the conditions by using higher concentrations of additional proteins in *λ*. These two experiments provide evidence that employing more concentration of proteins might be one of the ways that a cell uses to adapt itself in inhibiting conditions to facilitate mating. On the other hand, in Experiment 3 we look for specific proteins in *λ *that might be responsible for allowing a cell to change it response to pheromone from positive to negative. The results reveal that in some case the protein set *σ *(a subset of proteins in *λ*) is sufficient in regulating the response of the cell. In other cases, the requirements for the proteins in *σ *are more stringent. The Experiments 4, 5 and 6 are designed to study importance of different conditions for cell response. The results of these experiments show that there are certain conditions (edge weights) in the model that are more important in determining whether a cell will respond positively or not.

As a follow up of this work, we would like to probe more about the functionality of the proteins in set *λ*. In Experiment 3 we look at the performance of a subset of proteins (*σ*) in *λ*. In future work we plan to extend our simulation to individual proteins in the set *σ*. This can be done by isolating a particular protein and varying its available concentration in the simulations. There is possibility of future work for improving the model on several aspects. In our model the number of tokens exchanged during interaction of places and transitions are integers as ordinary Petri nets allow only that. However, in real life, the kd value of reactions cannot be always expected to be integral. We, therefore would like to modify our model so that it can handle the exchange of fractional tokens among its nodes. In the pheromone pathway, we have found evidence of negative feedback loops, which has not been implemented in our model. We plan to explore some other variant of Petri net which allows negative feedback loops. Finally, we would like to extend our work to other unicellular organisms apart from yeast, to study their pheromone pathways and try to identify possible simlarities between the pheromone pathway across species.

## Competing interests

The authors declare that they have no competing interests.

## Authors' contributions

AM implemented the model, carried out the experiments and drafted the manuscript. SDS and JSD partcipated in the design of experiments and data analysis and helped with the manuscript. SH offered crucial biological insight in the formulation of the model and experiments and served as the in house biological expert.

## Declarations

The research reported in this paper was in part supported by National Science Foundation grant CBET 1159933, National Science Foundation grant number 0743783 and by a University of Nebraska-Lincoln (UNL) grant, from UNL-Wheat, Wheat Products Research Program.

This article has been published as part of *BMC Bioinformatics *Volume 15 Supplement 7, 2014: Selected articles from the 10th Annual Biotechnology and Bioinformatics Symposium (BIOT 2013). The full contents of the supplement are available online at http://www.biomedcentral.com/bmcbioinformatics/supplements/15/S7
